# Microecological preparation combined with an modified low-carbon diet improves glucolipid metabolism and cardiovascular complication in obese patients

**DOI:** 10.1186/s13098-021-00697-6

**Published:** 2021-07-13

**Authors:** Jianguo Liu, Liehui Xiao, Hezhongrong Nie, Yong Pan, Yan Liu, Zhentian Zhang, Xiuping Lin, Yuan Zhang, Jinchuang Cai, Muxiu Yang, Yajing Liu, Leijun Zhang, Aimin Xu, Cuifeng Zhu

**Affiliations:** 1 Shenzhen Hospital of Southern Medical University, University of Hong Kong, Shenzhen, China; 2 Department of Pathophysiology, School of Basic Medical Sciences, Shenzhen University, Guangdong, Shenzhen, 518000 China; 3 Department of Medicine, University of Hong Kong, Shenzhen, Hong Kong China; 4 State Key Laboratory of Pharmaceutical Biotechnology, Shenzhen, Hong Kong China

**Keywords:** Obese, Microecological preparation modified low-carbon diet, Intestinal barrier function, Glucolipid metabolism, Cardiovascular complication

## Abstract

**Objective:**

To investigate the impact of microecological preparation combined with modified low-carbon diet on the glucolipid metabolism and cardiovascular complication in obese patients.

**Methods:**

From August 2017 to July 2020, 66 obese patients were recruited, and administrated with an modified low-carbon diet with (group A) or without (Group B) microecology preparation and a balanced diet in control group (group C) for 6 months. Meanwhile, 20 volunteers administrated with a balanced diet were recruited as the healthy control group (group D).

**Results:**

After 6-month intervention, obese subjects in group A and B showed significant improvement of body and liver fat mass, reduction of serum lipid levels, intestinal barrier function markers, insulin resistance index (IRI), high blood pressure (HBP) and carotid intima thickness, as compared with subjects in group C. More importantly, subjects in group A had better improvement of vascular endothelial elasticity and intimal thickness than subjects in group B. However, these intervention had no effect on carotid atherosclerotic plaque.

**Conclusion:**

Administration of microecological preparation combined with modified low-carbon diet had better improvement of intestinal barrier function, glucose and lipid metabolism, and cardiovascular complications than low-carbon diet in obese patients, but the effect of a simple low-carb diet on carotid atherosclerotic plaque need to be further addressed.

## Introduction

Due to the unhealthy lifestyle and nutrient overtake, obesity and its related cardiovascular complications have become one of key risk factors leading to human morbidity and mortality worldwide. It brings serious economic burden to patients, their families and the society [1, 2]. Previous studies have shown that the distribution of body fat in the Chinese population is characterized by central obesity [3]. Central obesity is one of the most important risk factors for chronic diseases such as cardiovascular disease and diabetes mellitus [4]. Therefore, losing weight is undoubtedly the effective outcome of the treatment of various metabolic diseases. To explore weight-loss methods that contribute to early glucose and lipid control is one of more important clinical significances in the evaluation of their safety and effectiveness.

There are many ways to lose weight clinically, such as fasting therapy, low carb diet therapy and ketogenic diet therapy [5]. Recent years, the most studied strategies for weight loss are the low carb diet. Several studies have shown that these nutritional approaches have beneficial effects and biochemical modification, and possess effective weight loss and cardiovascular risk parameters [6]. Furthermore, several studies have found that dietary therapy changed circulating lipid composition, represented by upregulation of short-chain fatty acids, and modulated cardiovascular homeostasis [6]. Although these methods have their own benefits, there is international debate over the adverse side effects. Therefore, seeking more safe and effective diet to lose weight has become a hot topic in the field of medical research.

Meanwhile gut micro-ecological imbalance is considered as a key factor leading to obesity and metabolic diseases [7]. Multiple clinical and basic studies have explored the effects and potential mechanisms in gut-cardiovascular axis [8]. Consistent to previous findings, our group also found that transplanting fecal bacteria from normal-weight mice to obese mice significantly reduced their body weight, glucolipid metabolism and insulin resistance [9]. However, these studies lack sufficient evidence for population application, and their mechanism of action remains to be explored.

In view of this, Our present study was to identify the therapeutic effects and safety of a weight-loss therapy using microecological preparation accompany with an modified low-carbon diet on the glucolipid metabolism and cardiovascular complication in obese patients.

## Materials and methods

### Selection of participants

Case collection and random grouping: from August 2017 to July 2020, 66 overweight or obese adult patients, aged between 18 and 60, were selected from the outpatient department of Nutrition and Health Management center of Shenzhen Hospital of Southern Medical University. They were divided into groups voluntarily and randomly, group A (N = 23): subjects were given intestinal microecological preparation + modified low-carbon diet; Group B (N = 23): subjects were given the diet with modified low carb diet; Group C (N = 20): subjects were given a balanced diet; In addition, 20 patients with normal physical examination were recruited and given a balanced diet (group D, N = 20).This study has been approved by the Ethics Committee of Shenzhen Hospital of Southern Medical University, and all the subjects signed an informed consent.

### Obesity diagnostic criteria and exclusion criteria

Diagnostic criteria and related definitions of diabetes and hypertension: diabetes was defined as fasting blood glucose (FBG) ≥ 7.0 mmol/L, 2-h postprandial blood glucose ≥ 11.1 mmol/L, or previous history of diabetes and currently being treated with hypoglycemic drugs. Hypertension was defined as systolic blood pressure (SBP) ≥ 140 mmHg and/or diastolic blood pressure (DBP) ≥ 90 mmHg, or a previous history of hypertension and currently being treated with antihypertensive medications.

### Experimental group setting

Group A: Obese subjects were treated with intestinal microecological preparation + modified low carb diet, three meals a day (total daily energy intake 1023.54–1093.54 kcal, protein intake 93.18–105.88 g, accounting for 36.41–38.73%, carbohydrate intake 48.29–60.09 g, accounting for 18.87–21.98%, fat intake 52.04–60.14 g, accounting for 45.76–49.50%), details are as follows:

Staple food: On the basis of strictly restricting the intake of all vegetables or starches containing more than 5% sugar, substitute nutrition bars for staple foods (purchased from Hangzhou saineng pharmaceutical company, containing 385.5 kcal, 28.8 g protein, 14.6 g fat, 34.7 g carbohydrate, 13.0 g dietary fiber and 515 mg sodium per 100 g).

Non-staple food: breakfast: 1 egg + 50 g dried tofu + 200 g vegetables such as tomato or cucumber, lunch and dinner: 50 g net meat + 80 g fish or shrimp + 200 g vegetables with less than 5% sugar, melon or algae vegetables.

Dietary supplements: [1] calcium: 300 mg (men) or 600 mg (women)/time, once a day, after lunch; (2) multi-dimensional element tablets (Wyeth shancun tablets), 1 tablet, once a day, after lunch; 3) ferrous succinate tablets 0.1 g(male) or 0.2 g(female)/time, 2 times a day, after breakfast and dinner; 4) sodium bicarbonate tablets 0.5 g/time, dissolved in 2000 ml warm water, 200 ml/h, orally divided into 10 times.

Intestinal microecological preparation: (1) compound glutamine enteric solution capsule (purchased from diao group chengdu pharmaceutical co., LTD., which was a compound preparation, including l-glutamine 1 g/piece), 2 tablets, 2 times a day, before breakfast and dinner; (2) bifidobacterium trifecta enteric solution capsule, which was a compound preparation, each gram contains long bifidobacterium ≥ 1.0 × 10^6^ CFU, lactobacillus acidophilus ≥ 4.0 × 10^6^ CFU, enterococcus faecalis ≥ 1.0 × 10^6^ CFU), 2 tablets, 2 times a day, eat after breakfast and dinner; (3) probiotics (ekeli) (purchased from Beijing tongze kangcheng medical technology co., LTD., ingredients: galactose oligosaccharide, trisaccharide and mannose oligosaccharide), 2 g/day, 1 time a day, breakfast; (4) the metabolite of lactic acid bacteria jk-21 (purchased from xiehe co., LTD., Japan, containing more than 1000 kinds of nucleic acid, amino acid, short-chain fatty acid, vitamin, trace mineral, sugar, etc. (analyzed by gas chromatography of institute of Japanese riken chemistry), resistant to gastric acid, high temperature), 1/4 strip/day, once a day, breakfast. No fruit or nuts, no coffee, no sugar or alcohol, no smoking during the intervention.

Exercise: moderate intensity aerobic exercise, 30–60 min daily exercise, daily exercise energy consumption of 300–500 kcal.

Group B: except not to eat intestinal microecological preparations, other diet, exercise and treatment plans were the same as group A; Group C: eat normally balanced diet (protein accounted for 10–15%, carbohydrate accounted for 55–60%, and fat accounted for 25–30%) according to the standard of 25–28 kcal/kg day, exercise was the same as group A. Group D: normal weight control group eat normally balanced diet as group C, exercise was the same as group A.

### Testing instrument or kit

Body fat was measured by body composition analyzer (bailida, #Mc-980a, TANITA, Tokyo, Japan); The detection instrument of glucose, lipid index was roche cobasc702, the kit was roche matching kit (Roche Diagnostics, Basel, Switzerland), and the detection method was colorimetry, electrochemiluminescence detection fasting insulin(FIns) (Roche Diagnostics, Basel, Switzerland), Fatty liver and atherosclerosis was evaluated by a Kelly S40 Plus color Doppler ultrasound device (probe frequency of 3.5 Hz); The intestinal barrier function measuring instrument and kit were purchased from Beijing zhongsheng jinyu diagnostic technology co., LTD.

Homeostasis model assessment of insulin resistance index (HOMA-IR) = FBG(mmol/L) × FINS(uU/m1)/22.5, When HOMA-IR ≥ 2. 69 was defined as insulin resistance [10].

### Results analysis

A database was established and the statistical analysis was performed by using SPSS 25.0 version. T-test was used for measurement data, person X^2^ test for counting data, rank sum test for rank data, and t-test was performed after correction for grade data. P < 0.05 was considered as significant difference.

## Results

### General information and intestinal barrier function

There was no significant difference among the general information such as age, gender, smoking, and alcohol intake (P > 0.05), but the intestinal barrier permeability and total daily energy intake in these three obese groups were obvious higher than that in the normal control group (P < 0.01 or P < 0.05), while the average daily physical activity was lower than that in the normal control group (P < 0.05) (see Table 1).

**Table 1:** Comparison of general data between groups before intervention (Mean ± SEM), Mean (95% CI)

	Group A	Group B	Group C	Group D	T value or Person X^2^	P value
Cases (male/female)	23 (13/10)	23 (10/13)	20 (9/11)	20 (9/11)	4.110	0.250
Age	37.22 ± 10.99	36.91 ± 10.78	37.05 ± 8.65	37.90 ± 11.40	t = 0.438	0.667
Smoker(%)	7 (30.43%)	6 (26.09%)	5 (25%)	5 (25%)	X^2^ = 0.227	0.973
Alcoholism(%)	14 (60.87%)^DDC^	13 (56.52%)^DDC^	10 (50%)	9 (45%)	X^2^ = 1.266	0.737
95%CI Routine exercises	913 (732, 1095)^DD^	978 (791, 1164)^DD^	953 (669, 1237)^DD^	1268 (1070, 1466)	t = 4.271	0.000
95%CI Food energy intake	2151 (2084, 2221)^DD^	2116 (2044, 2187)^DD^	2125 (2053, 2196)^DD^	1675 (1614, 1736)	t = 3.296	0.004
95%CI Protein to heat ratio	11.8 (11.7, 12.1)^DD^	12.5 (12.5, 12.6)^DD^	12.4 (12.3, 12.4)^DD^	18.5 (18.2, 18.7)	t = 3.455	0.003
95%CI Carbohydrate to heat ratio	53.8 (52.9, 54.7)	54.1 (53.2, 55.1)	54.3 (53.4, 55.2)	52.8 (51.6, 53.5)	t = 1.782	0.088
95%CI fat to heat ratio	34.6 (33.8, 35.4)^D^	33.5 (32.7, 34.3)^D^	33.7 (32.9, 34.6)^D^	29.0 (28.2, 30.8)	t = 2.606	0.017
HBP (%)	5 (21.74%)	3 (13.04%)	3 (15.00%)	0 (0.00%)	X^2^ = 4.673	0.197
Prevalence of abnormal glucose metabolism (%)	8 (34.78%)^DD^	7 (30.43%)^DD^	6 (30.00%)^DD^	0 (0.00%)	X^2^ = 8.589	0.035
Prevalence of carotid atherosclerotic plaque (%)	15 (65.22%)^DD^	14 (60.87%)^DD^	12 (60.00%)^DD^	0 (0.00%)	X^2^ = 23.883	0.000

T test was used for measurement data, and person X2 test was used for counting data. P < 0.05 was considered to be a significant difference.DD was compared with group D before intervention (P < 0.01), and D was compared with group D before intervention (P < 0.05).Comparison between BB and group B before intervention, P < 0.01; comparison between group B and group B before intervention, P < 0.05.CC was compared with group C before intervention (P < 0.01), and C was compared with group C before intervention (P < 0.05)

### Comparison of changes in body composition and glucolipid metabolism

As showed in Table 2, before intervention, body mass index, body fat, lipid level, FBG, FIns and HOMA-IR of three obese groups were significant higher than that of normal control group (P < 0.01 or P < 0.05). After 6 months of different diet and weight loss intervention, the body mass index, body fat, lipid level, FBG, FIns and HOMA-IR in group A and group B were decreased, as compared with group C (P < 0.01 or P < 0.05). More importantly, the improvement of these indicators in group A were significant better than that in group B (P < 0.01 or P < 0.05) (Table 2).

**Table 2:** Compared the changes of body composition such as body mass index, body fat and muscle mass in each group before and after intervention

	Group A (N = 23, M/F = 13/10)		Group B (N = 23, M/F = 10/13)		Group C (N = 20, M/F = 9/11)		Group D (N = 20, M/F = 9/11)	
	Before intervene	After intervenes	Before intervene	After intervenes	Before intervene	After intervenes	Before intervene	After intervenes
BMI (Kg/M2)	31.232 ± 1.10^DD^	26.793 ± 0.972^ddc^**	29.85 ± 0.77^DD^	27.06 ± 0.75^dd^*	30.30 ± 0.53^DD^	29.60 ± 0.51^dd^	22.57 ± 1.42	22.12 ± 0.32
WHR (W/H)	93.43 ± 1.88^D^	88.74 ± 1.72^dc^**	93.32 ± 1.37^D^	88.36 ± 1.31^dc^**	93.34 ± 1.03^D^	91.66 ± 1.25^d^	87.20 ± 0.88	86.44 ± 1.00
Fat Mass (kg)	32.30 ± 2.22^DD^	22.40 ± 1.90^cc^**	31.13 ± 1.74^DD^	24.90 ± 1.75^ccdd^*	31.05 ± 1.51^DD^	29.63 ± 1.49^dd^	16.00 ± 0.82	15.61 ± 0.78
Muscle Mass (kg)	51.22 ± 2.42^DD^	50.57 ± 2.34^d^	46.83 ± 1.87	45.27 ± 1.79	46.48 ± 1.95	46.04 ± 1.96	43.19 ± 1.60	42.40 ± 1.65
TG (mmol/L)	2.03 ± 0.31^D^	1.16 ± 0.11^c^*	2.09 ± 0.34^D^	1.34 ± 0.17*	2.22 ± 0.36^DD^	1.83 ± 0.27^dd^	1.06 ± 0.09	1.05 ± 0.06
TC (mmol/L)	6.25 ± 0.14^DD^	4.86 ± 0.14^ddcc^**	6.17 ± 0.12^DD^	5.21 ± 0.10^ddcc^**	6.33 ± 0.14 ^DD^	6.00 ± 0.12^dd^	4.30 ± 0.12	4.18 ± 0.10
HDLc (mmol/L)	1.44 ± 0.09	1.52 ± 0.07	1.42 ± 0.10	1.50 ± 0.07	1.39 ± 0.05	1.47 ± 0.10	1.42 ± 0.09	1.50 ± 0.09
LDLc (mmol/L)	4.41 ± 0.17^DD^	3.13 ± 0.13^dbcc^**	4.32 ± 0.13^DD^	3.47 ± 0.08^ddcc^**	4.55 ± 0.20^DD^	4.15 ± 0.13^dd^	2.76 ± 0.14	2.62 ± 0.10

The measurement data were tested by t test, and P < 0.05 was considered as significant difference. ** comparison before and after intervention, P < 0.01, * comparison before and after intervention, P < 0.05; DD was compared with group D before intervention (P < 0.01), and D was compared with group D before intervention (P < 0.05).Compared with group C before intervention, P < 0.05; Dd was compared with group D after intervention (P < 0.01), and D was compared with group D after intervention (P < 0.05). Cc was compared with group C after intervention (P < 0.01), and C was compared with group C after intervention (P < 0.05)

### The hepatic modification during intervention

**Fig. 1:**
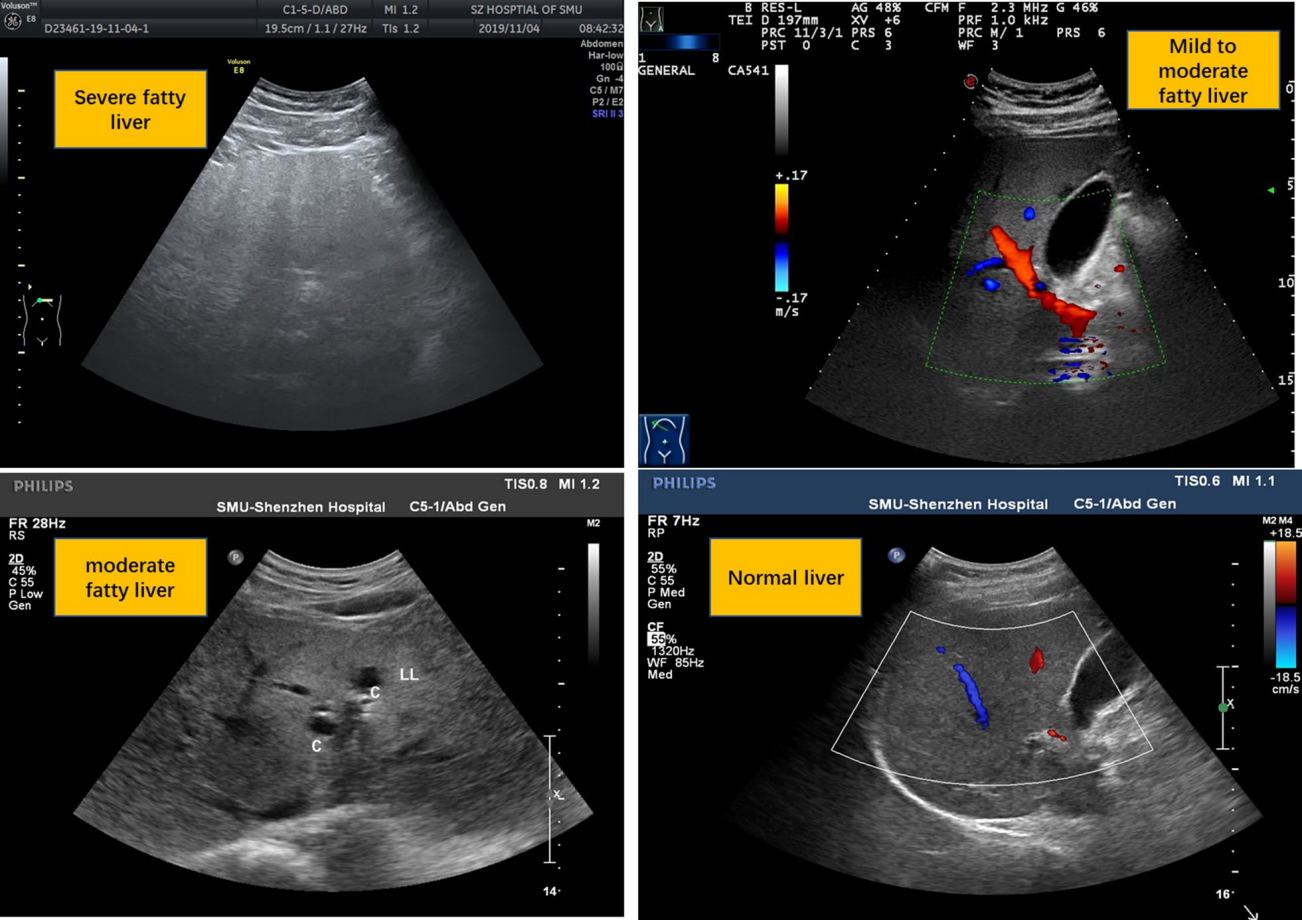
Ultrasonographic images of fatty liver at different degrees

Before intervention, the incidence and severity of fatty liver in obese patients were significant higher than normal control group (P < 0.01 or P < 0.05), After 6 months of different diet and weight loss intervention, the incidence and severity of fatty liver was obvious attenuated in group A, whichwas better than group B. The improvement of these hepatic modification in both A and B was more significant, as compared with group C (P < 0.01 or P < 0.05) (Table 3). Furthermore, as showed in Fig. 1, obese patients in group A had significant improvement of hepatic structure than group B and C.

**Table 3:** Comparison of the incidence and variation trend of fatty liver at different degrees before and after intervention in each group (component ratio %)

Prevalence of fatty liver (%)	Group A (N = 23, M/F = 13/10)		Group B (N = 23, M/F = 10/13)		Group C (N = 20, M/F = 9/11)		Group D (N = 20, M/F = 9/11)		
	Before intervene	After intervenes	Before intervene	After intervenes	Before intervene	After intervenes	Before intervene		After intervenes
Normal liver (%)	4 (17.39%)	9 (39.13%)	5 (21.74%)	8 (34.78%)	3 (15%)	3 (15%)	19 (95%)		19 (95%)
Mild fatty liver (%)	4 (17.39%)	4 (17.39%)	4 (17.39%)	5 (21.74%)	4 (20%)	5 (25%)	1 (5%)		1 (5%)
Moderately fatty liver (%)	5 (21.74%)	8 (34.78%)	5 (21.74%)	7 (30.43%)	5 (25%)	4 (20%)	0 (0%)		0 (0%)
Severe fatty liver (%)	10 (43.48%)	2 (8.70%)	9 (39.13%)	3 (13.04%)	8 (40%)	8 (40%)	0 (0%)		0 (0%)
Mann–Whitney rank sum test U value	Z_A-B_ = -0.369 Z_A-C_ = -0.090 Z_A-D_ = -4.966	Z_a-b_ = -0.26 Z_a-c_ = -2.077 Z_a-d_ = -3.841	Z_B-C_ = -0.280 Z_B-D_ = -4.736	Z_b-c_ = -1.823 Z_b-d_ = -4.047	Z_C-D_ = -4.967	Z_c-d_ = 0.000	Person X^2^ = 45.240		Person X^2^ = 31.402
Rank sum test, asymptotic significance P value	P_A-B_ = 0.712 P_A-C_ = 0.929 P_A-D_ = 0.000	P_a-b_ = 0.791 P_a-c_ = 0.038 P_a-d_ = 0.000	P_B-C_ = 0.779 P_B-D_ = 0.000	P_b-c_ = 0.068 P_b-d_ = 0.000	P_C-D_ = 0.000	P_c-d_ = 0.000	p = 0.000		p = 0.000

The Mann–Whitney rank sum test was used for the grade data. P < 0.05 was considered as significant difference. ** Before and after intervention group comparison, P < 0.01, * Before and after intervention group comparison, P < 0.05; Compared with group D before intervention, P < 0.01; compared with group D before intervention, P < 0.05; Compared with group D after dd intervention, P < 0.01; compared with group D after dd intervention, P < 0.05; After intervention, cc was compared with group C (P < 0.01). After intervention, cc was compared with group C (P < 0.05)

### The changes of blood pressure and cardiovascular complication during intervention

Before intervention, systolic blood pressure (SBP), diastolic blood pressure (DBP), carotid intima thickness and the incidence of carotid atherosclerotic plaque in three obese groups were significant higher than normal control group (P < 0.01 or P < 0.05), After 6 months of different diet and weight loss intervention, DBP, the carotid intima thickness and the incidence of carotid atherosclerotic plaque in group A was significantly decreased, as compared with group C (P < 0.01 or P < 0.05), but the incidence of carotid atherosclerotic plaque in group B were increased than group A and group C (P > 0.05) (Table 4).

**Table 4:** Comparison of the changes of intestinal barrier function in each group before and after intervention (Mean ± SEM)

Serum biomarker	Group A (N = 23, M/F = 13/10)		Group B (N = 23, M/F = 10/13)		Group C (N = 20, M/F = 9/11)		Group D (N = 20, M/F = 9/11)	
	Before intervene	After intervenes	Before intervene	After intervenes	Before intervene	After intervenes	Before intervene	After intervenes
d-lactic acid (mg/L)	15.73 ± 0.83^DD^	10.67 ± 0.67^ddbbcc^**	16.13 ± 0.91^DD^	14.30 ± 0.80^dd^	14.86 ± 0.94^DD^	13.82 ± 0.88^dd^	7.16 ± 0.39	6.19 ± 0.38
Diamine oxidase (U/L)	1.31 ± 0.07^DD^	2.51 ± 0.20^ddbbcc^**	1.56 ± 0.17^DD^	1.99 ± 0.20^dd^*	1.47 ± 0.15^DD^	1.87 ± 0.18^dd^	3.35 ± 0.20	3.43 ± 0.17
Serum LPS (U/L)	2.27 ± 0.25^D^	1.08 ± 0.13^bcc^**	2.41 ± 0.26^D^	1.92 ± 0.22^dd^	2.23 ± 0.25^D^	2.10 ± 0.22^dd^	1.29 ± 0.33	0.98 ± 0.28

The measurement data were tested by t test, and P < 0.05 was considered as significant difference. ** comparison before and after intervention, P < 0.01, * comparison before and after intervention, P < 0.05; DD was compared with group D before intervention (P < 0.01), and D was compared with group D before intervention (P < 0.05).Dd was compared with group D after intervention (P < 0.01), and D was compared with group D after intervention (P < 0.05).Comparison between bb and group B after intervention, P < 0.01; comparison between group B and group B after intervention, P < 0.05.Cc was compared with group C after intervention (P < 0.01), and C was compared with group C after intervention (P < 0.05)

## Discussion

Obesity has become a global public health problem, with 641 million adult obese in 2014 [11]. The number of obese people will increase to 1.1 billion by 2025 [2], and children and adolescents are also at high risk for overweight and obesity [12]. According to a report released by International organization for economic cooperation and development (OECD) October 2019, from analyzing 52 countries of overweight and obese subjects, the report predicted that if no effective measures are taken to reverse the trend of the development of obesity, overweight and obesity will make the life expectancy shortened about 3 years. Besides, these newly diagnosed subjects will develop into nearly 60 percent of new diabetes cases, 18 percent of cardiovascular disease, 11 percent of dementia and 8 percent of cancer [13–15]. Therefore, the prevention and treatment of obesity and related cardiovascular complications have become urgent global public health problems.

In recent years, many studies had proved that the low carb diet (carbohydrates consuming 50–150 g/day, energy ratio: 10–26%) and ketogenic diet (carbohydrate intake is less than 50 g/day, energy supply ratio < 10%) both have significant weight loss effect [16]. Studies have found that reducing carbohydrate intake can change the way of the body provides energy, from burning glucose to burning fat, which lowering insulin levels and playing a role in metabolic regulation [17–19]. However, more data are needed to support the safety and effectiveness of vitamin and trace element deficiency, as well as their effects on liver, kidney and cardiovascular functions, which often occur in the process of weight loss [20, 21]. Therefore, it is urgent to seek for a weight loss diet method that is safer, more effective, and has better compliance.

Obesity is one of risk factors, if left untreated, will often progress to greater metabolic defects, such as type 2 diabetes, nonalcoholic fatty liver disease, HBP, and coronary heart disease. Recent findings showed that there was close interplay of the intestinal microbiota with host metabolism and obesity [22]. Mechanistically, it has been mediated by many factors, including a defective gut barrier, bile acid metabolism, antibiotic use, and the pleiotropic effects of microbially produced metabolites. Several studies have hinted that high-fat diet can influence the abundance of intestinal flora and intestinal structure in mice, leading to obesity and insulin resistance [23]. These data showed that events that start in the gut, often in response to external cues such as diet and circadian disruption, have profound and lasting effects beyond the gut. In addition, there were lack sufficient evidence for population application, and their mechanism of action remains to be explored.

**Fig. 2:**
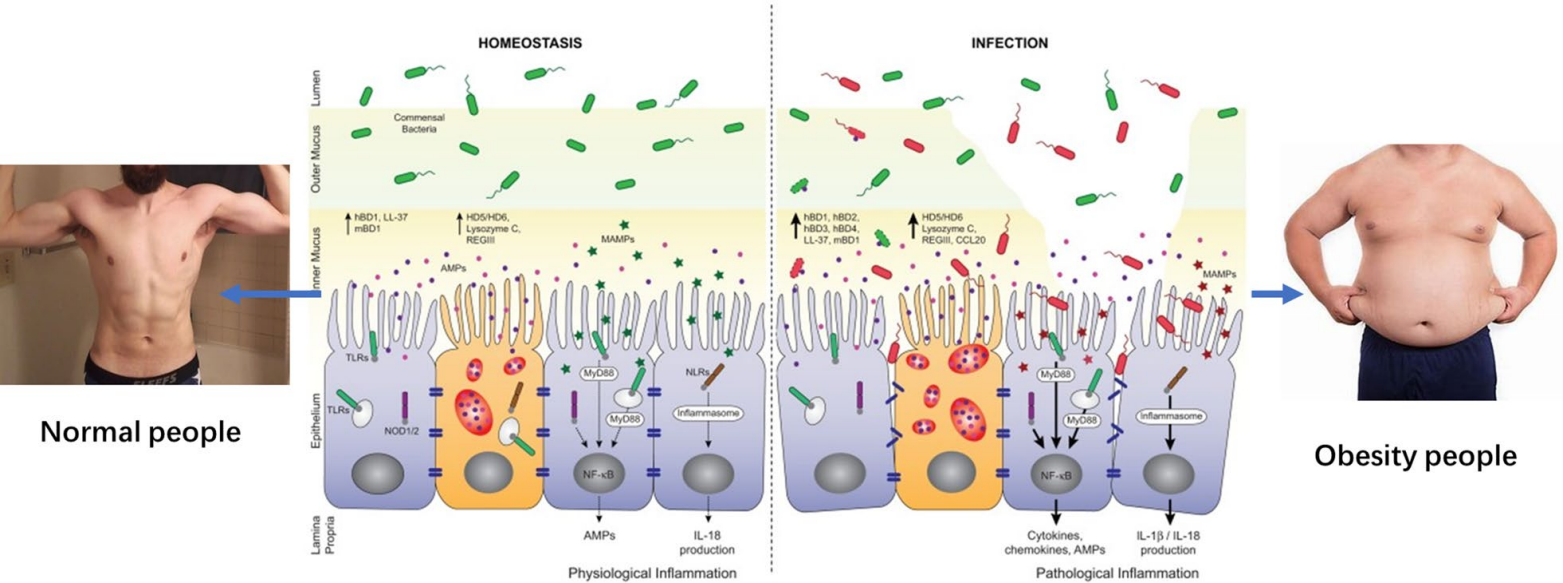
Schematic diagram of obesity and intestinal mucosal barrier dysfunction

In view of this, this study observed the application effect and security of a weight-loss therapy using microecological preparation (probiotics + prebiotics + metabolites of intestinal flora in combination)accompany with an modified low-carbon diet on the glucolipid metabolism and HBP, cardiovascular complication in obese patients. Our results found that these three obese patients had adverse clinical parameters, including total energy, sugar, carbohydrate, fat intake, and intestinal barrier parameters of diamine oxidase, permeability markers D-lactic acid, LPS, as compared with normal control group (Fig. 2). Consistent with our present study, a previous report found that low-grade inflammation was the hallmark of metabolic disorders, such as obesity, type 2 diabetes and nonalcoholic fatty liver disease [24]. Emerging evidence indicateed that these disorders were characterized by alterations in the intestinal microbiota composition and its metabolites, which translocated a disrupted intestinal barrier to affect various metabolic organs, such as the liver and adipose tissue. Thereby, it led to metabolic inflammation and abnormal body energy homeostasis. The present study proposed a concept by which the gut microbiota fuels metabolic inflammation and dysregulation.

For many years, food and nutritional intervention studies have been concentrated on reducing dietary fat, but there was little positive outcomes over the long-term. Recent years, the most studied strategies for weight loss are the low carb diet and ketogenic diet. Many studies have shown that these nutritional approaches have different levels of physiological and biochemical basis, and possess effective weight loss accompany with improvement of cardiovascular risk parameters [25]. Similarly, our results showed the clinical parameters, including the body mass index, body fat, FBG, FIns, HOMA-IR, lipid level, incidence and severity of fatty liver, systolic pressure and diastolic pressure in group A and group B were significantly improved, as compared with group C after 6 months of different diet and weight loss intervention. The improvement effect of carotid intima thickness and the incidence of carotid atherosclerotic plaque in group A was also more significant than group D (normal group). These results was consistent with the previous findings, which indicated that there was close interaction between CVD and deleterious alterations of gut microbiota [26]. Recent data indicated that microbial metabolites, as well as structural bacterial components, could migrate from the intestinal environment to the general circulation, where they interacted and modified the function of relevant tissues [27]. Acetate (C2), propionate (C3), and butyrate (C4) are the most abundant short-chain fatty acids (SCFA) and comprise 95% of all those found in the body which produced by the gut microbiota as byproducts of fermentation of dietary fibers. These SCFA are found in the intestines and have multiple effects within the gastrointestinal tract, including stimulation of ileal motility and mucus production, maintenance of epithelial health by up-regulating the expression of tight junction proteins, and as a major fuel source for colonic epithelial cells [28]. Although the majority of SCFA are metabolized within the colon, a small percentage are absorbed and found in the systemic circulation. Several studies have shown that SCFA released in the systemic circulation have modulatory effects on cardiovascular function [29]. Furthermore, Several studies have shown that SCFA that reach the systemic circulation have modulatory effects on cardiovascular function. For example, administration of acetate or butyrate have been shown to reduce blood pressure in experimental models of hypertension [30]. In the present study, we also supported administration of SCFA in obese subjects had beneficial effects on metabolic parameters.

Our present study identified a unique formulation composition, which contains both beneficial bacteria and prebiotics to prevent intestinal toxic pool overflow. After uptake, it can be quickly and comprehensively metabolized to probiotics. It then stabilized intestinal immunity and maintained normal intestinal function through intestinal microecological optimization. The metabolites of intestinal flora contained metabolites such as p-aminobutyric acid and SCFA. Several studies have confirmed that probiotics can enhance the function of tight connection of mucosal barrier [31]. In addition, probiotics can affect the physiological process of intestinal mucosal epithelial cells through binding pattern recognition molecules and toll-like receptors, thus stimulate the activation of macrophages in the mucosal barrier. Probiotics can also modulate the immune response of intestinal dendritic cells, B and T cells, thereby reducing the metabolic inflammatory response [32]. However, consuming probiotics alone to achieve weight loss is not sufficient, because there are many factors that influence body fat composition, including genetic inheritance, age, exercise [33, 34]. Therefore, dietary fiber as raw materials with different structure of prebiotics added preparations, and intestinal flora metabolism product such as short chain fatty acid and amino acid metabolites (specific neurotransmitters, such as gamma-aminobutyric acid, serotonin, and nitric oxide), can improve whole body metabolism through a variety of ways, such as the gut hormone glucagon-like peptide (GLP)-1, blood sugar and inflammation [35]. Besides, specific bacterial components such as ClpB and Amuc_1100 can regulate dietary intake, maintain intestinal barrier function, and ultimately control host metabolism [36, 37]. In addition, our compound intestinal microecological preparation contains compound glutamine enteric solution capsules. Studies have proved that glutamine can improve the absorption, secretion and motor function of the intestine, and promote the replacement of damaged intestinal mucosa [38].

In the present study, there was a limitation. Even though we monitored the clinical parameters every 2 weeks, it still difficult to know how much the patients actually followed a low carb diet or followed the directions proposed in the 6-month follow-up, which might bring some mistakes. In the future, we need to establish a clinical trial in obese subjects who housing in hospital for 6-month.

## Conclusion

It is one of the important pathogenesis of simple obesity that the unreasonable diet structure leads to the damage of intestinal barrier function and induces low level inflammatory response. The mechanism of microecological preparation combined with modified low-carbon diet improved intestinal barrier function, glucose and lipid metabolism and cardiovascular complications more effectively than low-carbon diet only in obese patients may be by regulating the enteral-brain axis signaling pathway. The effect of a simple low-carb diet on carotid atherosclerotic plaque deserves further attention.

## Data Availability

The data and materials will be provided if request.
